# Metabolic crossroads: unravelling immune cell dynamics in gastrointestinal cancer drug resistance

**DOI:** 10.20517/cdr.2024.164

**Published:** 2025-02-08

**Authors:** Chahat Suri, Babita Pande, Lakkakula Suhasini Sahithi, Shashikant Swarnkar, Tuneer Khelkar, Henu Kumar Verma

**Affiliations:** ^1^Department of Oncology, Cross Cancer Institute, University of Alberta, Edmonton AB T6G 1Z2, Canada.; ^2^Department of Physiology, All India Institute of Medical Sciences, Raipur 492099, India.; ^3^Department of Biotechnology, Guru Ghasidas Vishwavidyalaya, Bilaspur 495009, India.; ^4^Department of Biochemistry, C.C.M. Medical College, Bhilai 490020, India.; ^5^Department of Botany and Biotechnology, Govt. Kaktiya P G College, Jagdalpur 494001, India.; ^6^Department of Immunopathology, Institute of Lung Health and Immunity, Comprehensive Pneumology Center, Helmholtz Zentrum, Munich 85764, Germany.

**Keywords:** Gastrointestinal cancers, immune cells, tumor microenvironment, metabolic pathways, drug resistance

## Abstract

Metabolic reprogramming within the tumor microenvironment (TME) plays a critical role in driving drug resistance in gastrointestinal cancers (GI), particularly through the pathways of fatty acid oxidation and glycolysis. Cancer cells often rewire their metabolism to sustain growth and reshape the TME, creating conditions such as nutrient depletion, hypoxia, and acidity that impair antitumor immune responses. Immune cells within the TME also undergo metabolic alterations, frequently adopting immunosuppressive phenotypes that promote tumor progression and reduce the efficacy of therapies. The competition for essential nutrients, particularly glucose, between cancer and immune cells compromises the antitumor functions of effector immune cells, such as T cells. Additionally, metabolic by-products like lactate and kynurenine further suppress immune activity and promote immunosuppressive populations, including regulatory T cells and M2 macrophages. Targeting metabolic pathways such as fatty acid oxidation and glycolysis presents new opportunities to overcome drug resistance and improve therapeutic outcomes in GI cancers. Modulating these key pathways has the potential to reinvigorate exhausted immune cells, shift immunosuppressive cells toward antitumor phenotypes, and enhance the effectiveness of immunotherapies and other treatments. Future strategies will require continued research into TME metabolism, the development of novel metabolic inhibitors, and clinical trials evaluating combination therapies. Identifying and validating metabolic biomarkers will also be crucial for patient stratification and treatment monitoring. Insights into metabolic reprogramming in GI cancers may have broader implications across multiple cancer types, offering new avenues for improving cancer treatment.

## INTRODUCTION

Gastrointestinal cancers (GI) encompass a diverse group of malignancies affecting the digestive tract, including cancers of the esophagus, stomach, liver, pancreas, colon, and rectum. These cancers collectively represent a significant global health burden^[1,2]^. According to the latest data from the Global Cancer Observatory (GLOBOCAN) 2023, GI cancers account for approximately 26% of all new cancer cases and 35% of all cancer-related deaths worldwide^[3]^. Colorectal cancer ranks as the third most commonly diagnosed cancer and the second leading cause of cancer death globally, with nearly 2 million new cases and over 900,000 deaths reported annually^[4]^. Gastric and liver cancers also remain among the top causes of cancer mortality, particularly in regions like East Asia and sub-Saharan Africa, where risk factors such as *Helicobacter pylori* infection and hepatitis B virus (HBV) are prevalent^[5]^. Despite advancements in early detection, surgical techniques, and therapeutic approaches, the prognosis for many GI cancers remains poor, especially in advanced stages. Standard treatment modalities, including surgery, chemotherapy, radiation therapy, and targeted therapies, have improved outcomes for some patients. However, the development of drug resistance, where cancer cells adapt and survive despite treatment, continues to pose a significant challenge^[6,7]^. This resistance not only limits the effectiveness of existing therapies but also contributes to disease recurrence and progression, underscoring the urgent need for novel treatment strategies^[8]^. The historical timeline of GI Cancer research and immune drug resistance is highlighted in Table 1.

**Table 1:** Historical timeline for gastrointestinal cancer research and immune drug

**1900s^[9]^**	· Early research in oncology begins to identify certain cancers, including GI, that have genetic and environmental causes · Early surgical interventions and basic chemotherapeutic agents are developed
**1950s^[10]^**	· The concept of immunotherapy begins to take shape with early studies suggesting the immune system’s role in cancer · At the same time, the development of chemotherapeutic agents like 5-FU starts, which later becomes a mainstay in treating GI cancers
**1960-1970s^[11]^**	· Development of more refined surgical techniques for GI cancers, such as improved methods for gastric and colorectal cancer resection · Introduction of radiation therapy as a standard treatment modality for GI cancers, improving local control of the disease
**1980s^[12]^**	· Research into monoclonal antibodies begins, leading to the first targeted therapies. Early studies on immune system modulation and its potential role in cancer treatment start to emerge
**1990s^[13,14]^**	· Identification of key molecular targets in cancer, such as HER2/neu in breast cancer, leads to targeted therapies like trastuzumab, which sets the stage for similar approaches in GI cancers · Introduction of interferon-alpha and interleukin-2 as immune-modulating agents, early use of immunotherapy in treating cancers including melanoma and renal cell carcinoma
**2000s^[15-17]^**	· 2010: FDA approval of bevacizumab (Avastin) for metastatic colorectal cancer, a targeted therapy that inhibits angiogenesis · 2011: Introduction of immune checkpoint inhibitors, such as pembrolizumab (Keytruda) and nivolumab (Opdivo), for various cancers including GI cancers, demonstrating significant clinical efficacy · 2015: Emergence of the concept of TMB and MSI as biomarkers for predicting response to immunotherapy
**2020s^[18,19]^**	· 2020: The FDA approves several new immune checkpoint inhibitors and combination therapies for GI cancers, including regorafenib and cabozantinib · 2021: Advances in understanding the role of the TME and its metabolic effects on immune response lead to new strategies for overcoming resistance to immunotherapy · 2022: The development of novel agents targeting metabolic pathways in immune cells and their role in cancer drug resistance is an active area of research · 2023: Research focuses on integrating multi-omics approaches (genomics, proteomics, metabolomics) to personalize treatment and enhance the efficacy of immunotherapies in GI cancers

GI: Gastrointestinal cancers; 5-FU: 5-fluorouracil; FDA: Food and Drug Administration; TMB: tumor mutational burden; MSI: microsatellite instability; TME: tumor microenvironment.

The TME plays a crucial role in the initiation, progression, and therapeutic response of GI cancers^[20]^. The TME is a complex and dynamic milieu composed of various cell types, including cancer cells, stromal cells, endothelial cells, and, importantly, immune cells. These immune cells - such as tumor-associated macrophages (TAMs), myeloid-derived suppressor cells (MDSCs), regulatory T cells (Tregs), and others are the key components of the TME^[21]^. They interact closely with cancer cells and contribute to an immunosuppressive environment that facilitates tumor growth and protects the tumor from immune-mediated destruction. The crosstalk between cancer cells and immune cells within the TME is mediated by a variety of signaling molecules, cytokines, and metabolic exchanges. These interactions can lead to immune evasion, where cancer cells escape detection and elimination by the immune system, and can promote resistance to therapies, including chemotherapy, targeted therapies, and immunotherapies^[22,23]^. Therapies targeting the dynamic metabolism have also proven to be more effective in this scenario^[23]^. Metabolic reprogramming is a hallmark of cancer, characterized by the alteration of metabolic pathways to support the high energy and biosynthetic demands of rapidly proliferating tumor cells^[24]^. However, this phenomenon is not limited to cancer cells alone. Immune cells within the TME also undergo significant metabolic changes that influence their function and behavior^[25]^. These metabolic adaptations in immune cells are critical for their survival and activity in the nutrient-deprived and hypoxic conditions of the TME^[26]^. In GI cancers, metabolic reprogramming of immune cells can contribute to tumor growth and the development of drug resistance^[27]^. TAMs may shift toward oxidative phosphorylation (OXPHOS) and fatty acid oxidation (FAO), promoting an anti-inflammatory and pro-tumorigenic phenotype^[28]^. Similarly, MDSCs and Tregs can enhance glycolysis and lipid metabolism, leading to immune suppression and decreased efficacy of anti-cancer therapies^[29]^. These metabolic alterations not only support the survival and function of immune cells within the TME but also play a pivotal role in the development of resistance to cancer therapies. Consequently, targeting the metabolic pathways of immune cells offers a promising strategy to overcome drug resistance and improve therapeutic outcomes in patients with GI cancers. This review will explore the intricate relationship between immune cell metabolism and drug resistance in GI cancers, discussing the underlying mechanisms and potential therapeutic implications.

## METABOLIC REPROGRAMMING IN THE TUMOR MICROENVIRONMENT

Metabolic reprogramming in the tumor microenvironment (TME) has emerged as a critical factor influencing cancer progression and therapeutic responses^[30]^. Recent studies have shed light on the complex interplay between cancer cells, immune cells, and stromal components within the tumor ecosystem^[31]^. A 2023 review by Jin *et al*. highlighted how lipid metabolic reprogramming affects not only tumor cells but also stromal and immune cells, shaping the immunosuppressive landscape of the TME^[30]^. The dynamic nature of metabolic alterations in tumors was emphasized in a 2022 study by Navarro *et al*., which described how cancer cells adapt to nutrient-poor and hypoxic conditions through various metabolic pathways^[32]^. De Martino *et al*. further elaborated on how these metabolic changes in cancer cells influence the recruitment and function of immune cells, potentially offering new therapeutic targets^[33,34]^. The impact of metabolic reprogramming on immune suppression was extensively discussed by Arner and Rathmell, who explored how altered metabolism in the TME affects T cell function and antitumor immunity^[34]^. A 2024 study provided insights into how metabolic alterations in colorectal cancer specifically influence the immune microenvironment, highlighting the potential for metabolism-targeted therapies^[35]^. Collectively, these recent works underscore the critical role of metabolic reprogramming in shaping the TME and its potential as a therapeutic target in cancer treatment.

Targeting the metabolism of cancer has a lot of potential, but it is difficult since cancer cells can quickly rewire their metabolic pathways in response to therapeutic treatment. Because of this metabolic flexibility, cancer cells can alternate between FAO, glycolysis, and other metabolic processes to meet their energy needs and continue to exist while undergoing treatment. Therefore, monotherapies that target specific metabolic enzymes or pathways frequently result in limited efficacy and the development of resistance. Potential approaches to lessen this adaptive resistance include combining metabolic inhibitors with conventional treatments like chemotherapy or immunotherapy, or utilizing combination medicines that target several metabolic pathways.

## IMMUNE CELL METABOLISM IN GI CANCERS

The TME in GI cancers represents a complex ecosystem comprising cancer cells, stromal cells, and diverse immune cell populations. Within this intricate milieu, the metabolic reprogramming of immune cells emerges as a critical factor influencing tumor progression and therapeutic responses^[36]^. As cancer cells proliferate rapidly and alter their surrounding environment, immune cells are forced to adapt to challenging conditions, including nutrient deprivation, hypoxia, and acidosis^[37]^. Rapid tumor growth outpaces blood vessel formation, leading to areas of low oxygen availability. This hypoxic condition prompts cancer cells and stromal cells, particularly cancer-associated fibroblasts, to adapt metabolically by enhancing glycolytic pathways and producing angiogenic factors to promote vascularization^[11]^. As tumors grow, they consume available nutrients such as glucose and amino acids at an accelerated rate. This depletion affects not only the tumor cells but also the surrounding immune cells such as macrophages, which may struggle to access essential nutrients for their activation and function^[34]^. The accumulation of metabolic by-products like lactate leads to a decrease in pH within the TME, creating an acidic environment that can inhibit immune cell function and promote tumor progression^[38]^. These adaptations profoundly impact the function and behavior of various immune cell populations, ultimately shaping the course of the disease and the efficacy of cancer treatments^[39]^. One of the major adaptations of immune cells is effector T cells switching from OXPHOS to glycolysis, which is less efficient but allows for rapid ATP production under nutrient-limited conditions^[40]^. The altered metabolism within the TME can also lead to an immunosuppressive environment. Tregs and MDSCs are often favored under these conditions, further inhibiting antitumor immune responses^[41]^. Moreover, cytotoxic lymphocytes may lose their ability to effectively kill tumor cells, while macrophages may adopt a pro-tumorigenic M2 phenotype instead of a tumor-killing M1 phenotype.

One of the primary challenges faced by immune cells in the TME is nutrient deprivation^[42]^. Cancer cells, particularly those exhibiting the Warburg effect, consume vast amounts of glucose, creating a competitive environment where immune cells struggle to meet their metabolic needs^[43]^. This glucose deprivation forces T cells and other immune cells to alter their metabolic profiles, often resulting in impaired effector functions. CD8+ T cells, which normally rely heavily on glycolysis for their cytotoxic activities, shift toward FAO to survive in the glucose-depleted environment^[44]^. While this metabolic flexibility enables survival, it often comes at the cost of reduced antitumor efficacy. The competition for nutrients extends beyond glucose, with cancer cells also depleting essential amino acids and lipids, further compromising immune cell function^[45]^.

Hypoxia represents another significant challenge in the TME of GI cancers. Poorly vascularized regions of tumors create oxygen-deprived areas, triggering metabolic adaptations in immune cells mediated largely by hypoxia-inducible factor 1α (HIF-1α)^[46]^. This transcription factor promotes glycolysis and suppresses OXPHOS, fundamentally altering cellular energy production^[47]^. The impact of hypoxia is particularly evident in TAMs, which often shift toward an M2-like phenotype in oxygen-poor regions, promoting immunosuppression^[48]^. This metabolic reprogramming in TAMs is associated with increased expression of arginase-1 and indoleamine 2,3-dioxygenase (IDO), enzymes that deplete essential amino acids required for T cell function^[49]^. The hypoxic environment also affects other immune cell populations, including dendritic cells and T cells, often impairing their antitumor functions and promoting a more immunosuppressive TME^[50]^. The accumulation of metabolic by-products, particularly lactic acid from cancer cell glycolysis, creates an acidic TME that further modulates immune cell function^[51]^. This acidic environment can impair the activity of effector T cells while promoting the differentiation and function of immunosuppressive cell types like Tregs^[52]^. The high lactate levels in the TME not only contribute to acidity but also serve as an alternative fuel source for some immune cells, particularly Tregs, further enhancing their immunosuppressive functions^[53]^. Moreover, the acidic environment affects the metabolism of dendritic cells, impairing their ability to present antigens and activate T cells effectively, thus compromising the initiation of antitumor immune responses^[54]^.

The impact of these metabolic challenges on specific immune cell populations in GI cancers is profound and varied. CD8+ T cells, crucial for antitumor immunity, often exhibit metabolic exhaustion due to chronic antigen stimulation and nutrient competition^[55]^. This exhaustion is characterized by reduced glycolytic capacity, mitochondrial dysfunction, and increased expression of inhibitory receptors like PD-1, collectively impairing their ability to combat tumor cells^[56]^. Natural killer (NK) cells, another vital component of antitumor immunity, show altered metabolism in the TME, with reduced glycolysis and OXPHOS contributing to their dysfunction and diminished cytotoxicity against cancer cells^[57]^. These metabolic alterations in NK cells compromise their ability to recognize and eliminate cancer cells, further contributing to tumor immune evasion in GI cancers.

The MDSCs and Tregs, both key players in tumor-induced immunosuppression, undergo significant metabolic reprogramming that enhances their immunosuppressive functions^[58]^. MDSCs increase their glycolytic activity and production of lactate, contributing to the acidic TME, while also enhancing arginine metabolism and FAO to support their survival and suppressive activities. Tregs, on the other hand, exhibit increased reliance on OXPHOS and FAO, allowing them to thrive in the glucose-depleted TME^[59,60]^. Their enhanced ability to utilize lactate as an energy source gives them a competitive advantage in an acidic environment, further promoting their immunosuppressive functions^[61]^. The metabolic reprogramming in immune cells of the TME is summarized in Table 2.

**Table 2:** Metabolic reprogramming in immune cells of the tumor microenvironment

**Cell type**	**Dominant metabolic pathway**	**Functional impact**	**Therapeutic target**	**References**
TAMs	FAO	Immunosuppression, tumor promotion	FAO inhibitors	[62]
CAFs	Glycolysis, glutaminolysis	Tumor growth support, immunosuppression	Glycolysis inhibitors, glutaminase inhibitors	[63]
Exhausted CD8+ T cells	Impaired glucose uptake, reduced mitochondrial function	Decreased antitumor activity	Metabolic reprogramming agents	[64]
MDSCs	Glycolysis, amino acid metabolism	T cell suppression, tumor progression	Glycolysis inhibitors, amino acid metabolism modulators	[65,66]
Tregs	FAO, glycolysis	Immunosuppression, inhibition of effector T cells	FAO inhibitors, glycolysis inhibitors	[30,67]

TAMs: Tumor-associated macrophages; FAO: fatty acid oxidation; CAFs: cancer-associated fibroblasts; MDSCs: myeloid-derived suppressor cells; Tregs: regulatory T cells.

## MECHANISMS OF DRUG RESISTANCE

Metabolic reprogramming in immune cells within the TME has emerged as a critical factor contributing to drug resistance in GICs. This complex interplay between altered cellular metabolism and immune function facilitates cancer cell survival and therapeutic evasion through several interconnected mechanisms^[68,69]^. In GI cancers, including colorectal, gastric, pancreatic, and esophageal malignancies, the tumor TME is characterized by nutrient deprivation, hypoxia, and acidosis. These conditions compel both cancer and immune cells to adapt their metabolic profiles, often with profound consequences for tumor growth and treatment efficacy^[70,71]^. One of the primary mechanisms by which metabolically reprogrammed immune cells contribute to drug resistance is through immune evasion^[72]^. TAMs, Tregs, and MDSCs adopt immunosuppressive phenotypes that inhibit antitumor immune responses^[73]^. These cells secrete factors such as interleukin-10 (IL-10), TGF-β, and prostaglandin E2, which suppress the activity of cytotoxic T lymphocytes and NK cells^[74]^. Moreover, increased FAO in Tregs enhances their immunosuppressive capacity, allowing cancer cells to evade immune surveillance and resist immunotherapies. This metabolic adaptation in immune cells creates a protective environment for cancer cells, shielding them from both endogenous immune responses and exogenous therapeutic interventions^[75]^. The resulting immunosuppressive TME not only promotes tumor growth but also diminishes the efficacy of various cancer treatments, including immunotherapies and some chemotherapeutic agents that rely on an intact immune response for optimal efficacy^[76]^.

Another significant mechanism of drug resistance involves the alteration of drug metabolism by immune cells within the TME^[77]^. Metabolically reprogrammed immune cells can express enzymes that metabolize and inactivate chemotherapeutic agents, reducing their efficacy^[78]^. Tumor-associated neutrophils have been shown to express high levels of aldehyde dehydrogenase, which can detoxify certain chemotherapy drugs like cyclophosphamide^[79]^. Similarly, increased expression of cytochrome P450 enzymes in tumor-infiltrating immune cells can lead to enhanced metabolism of various targeted therapies, limiting their effectiveness^[80]^. This metabolic alteration in immune cells contributes to the development of chemoresistance in various cancer types, highlighting the importance of considering the TME as a whole when designing treatment strategies^[81,82]^. Furthermore, the competition for essential nutrients between cancer cells and immune cells in the TME can significantly impact drug efficacy^[83]^. Cancer cells often outcompete T cells for glucose, leading to T cell dysfunction and impaired antitumor immunity. This nutrient deprivation can also limit the availability of metabolites required for optimal drug action, thereby contributing to therapeutic resistance^[84]^. The altered metabolic landscape within the TME can affect drug distribution, uptake, and activity, further complicating treatment efficacy^[85]^.

Metabolic pathways such as pyrimidine metabolism, lipid metabolism, glucose metabolism, and FAO have been implicated in the development of resistance to commonly used chemotherapeutic agents. For instance, 5-fluorouracil (5-FU) targets pyrimidine metabolism by inhibiting thymidylate synthase, yet cancer cells can upregulate this enzyme to mitigate drug effects^[86]^. Similarly, oxaliplatin, a platinum-based drug, encounters resistance through alterations in lipid metabolism that enhance cell survival during treatment^[87]^. Irinotecan, a topoisomerase I inhibitor, exemplifies how enhanced DNA repair mechanisms can enable cancer cells to recover from drug-induced damage. The competition for glucose between cancer cells and immune cells further complicates treatment efficacy, as nutrient deprivation can impair antitumor immune responses^[88,89]^.

The production of specific metabolites by reprogrammed immune cells can directly influence cancer cell behavior and drug response^[90]^. Lactate produced by glycolytic MDSCs can activate signaling pathways in cancer cells that promote survival and drug resistance. Additionally, metabolic alterations in immune cells can lead to changes in epigenetic regulation, affecting gene expression patterns that influence drug sensitivity^[91]^. Accumulation of the oncometabolite 2-hydroxyglutarate in IDH-mutant tumors has been shown to alter DNA methylation patterns, potentially contributing to therapeutic resistance^[92]^. These metabolite-mediated effects underscore the complex interplay between cellular metabolism and signaling pathways in the TME, which can collectively contribute to drug resistance mechanisms^[56]^. Moreover, changes in cellular redox balance resulting from metabolic reprogramming in immune cells can affect the efficacy of therapies that rely on oxidative stress induction. Enhanced glutathione metabolism in TAMs can protect cancer cells from oxidative damage induced by certain chemotherapies^[93]^.

Despite advances in treatment modalities, drug resistance remains a significant challenge, limiting therapeutic efficacy and impacting patient outcomes. 5-FU, a pyrimidine analog, has been a cornerstone of GI cancer treatment for decades, acting as an antimetabolite that inhibits thymidylate synthase and disrupts DNA synthesis^[94]^. Three major mechanisms contributing to 5-FU resistance include metabolic reprogramming, enhanced DNA repair mechanisms, and autophagy activation. Cancer cells upregulate thymidylate synthase expression, counteracting 5-FU’s inhibitory effects, and alterations in pyrimidine metabolism pathways further contribute to resistance^[95]^. Oxaliplatin, another commonly used drug in colorectal cancer combination therapies, is a platinum-based compound that forms DNA adducts, leading to cell cycle arrest and apoptosis^[96]^. Recent studies indicate that changes in lipid metabolism, particularly increased Fatty acid synthesis (FAS), contribute to oxaliplatin resistance in colorectal cancer cells^[97]^.

Irinotecan, the third most common drug for treating GI cancers, is a topoisomerase I inhibitor widely used for metastatic colorectal cancer. It induces DNA damage by preventing the relegation of single-strand breaks^[98]^. Irinotecan resistance often arises from topoisomerase I alterations, increased drug efflux, enhanced DNA repair, or disrupted apoptosis. Oxaliplatin resistance is linked to improved DNA adduct repair, glutathione detoxification, and altered drug accumulation^[99]^. Other drugs used for GI cancer treatment include gemcitabine and bevacizumab^[100]^. Resistance to bevacizumab, an anti-angiogenic agent, can arise through alternative angiogenic pathways where cancer cells activate other pro-angiogenic factors, such as fibroblast growth factor (FGF) and platelet-derived growth factor (PDGF), bypassing VEGF inhibition^[70]^. For gemcitabine, overexpression of ribonucleotide reductase subunits (RRM1 and RRM2) can counteract its inhibitory effects on DNA synthesis^[101]^. A 2022 study by N’Guessan *et al*. demonstrated that Combination therapy with saposin C-dioleoylphosphatidylserine (SapC-DOPS) and gemcitabine or Abraxane/GEM significantly inhibits tumor growth and improves survival in subcutaneous and orthotopic PDAC models compared to individual treatments^[102]^, leading to increased apoptosis, reduced tumor growth, and improved survival in preclinical models^[103]^.

Although less extensively studied than lactate dehydrogenase (LDH) inhibitors, GLUT-1 (glucose transporter-1) inhibitors also show promise in overcoming chemotherapy resistance in GI cancers. BAY-876, a GLUT-1 inhibitor, demonstrated synergistic effects with 5-FU in colorectal cancer models, potentially by disrupting the metabolic adaptations that support drug resistance^[104]^. Additionally, fasentin, a glucose uptake inhibitor, has shown the ability to sensitize resistant cancer cells to various chemotherapeutic agents, including gemcitabine, by limiting their metabolic flexibility^[105]^. However, as with targeted therapies, cancer cells may develop resistance to LDH and GLUT-1 inhibitors over time. The potential drug-resistance agents targeting GI cancers are listed in Table 3.

**Table 3:** Drugs targeting metabolic pathways in gastrointestinal cancers

**Drug**	**Mechanism of action**	**Metabolic pathway targeted**	**Cancer type**	**Status**
Gemcitabine	Nucleoside analog, DNA synthesis inhibitor	Pyrimidine metabolism	Pancreatic, biliary tract	FDA approved^[106]^
5-FU	Pyrimidine analog, thymidylate synthase inhibitor	Pyrimidine metabolism	Colorectal, gastric, pancreatic	FDA approved^[107]^
Oxaliplatin	DNA crosslinking agent	DNA repair, cellular stress response	Colorectal	FDA approved^[108]^
Irinotecan	Topoisomerase I inhibitor	DNA replication, cellular stress response	Colorectal	FDA approved^[109]^
Bevacizumab	VEGF inhibitor	Angiogenesis, glucose and oxygen metabolism	Colorectal	FDA approved^[110]^
Metformin	AMPK activator, mTOR inhibitor	Glucose metabolism, mitochondrial complex I inhibition	Colorectal, pancreatic	Clinical trials^[111]^
2-deoxyglucose	Glycolysis inhibitor	Glucose metabolism	Colorectal, gastric	Preclinical^[112]^
Dichloroacetate	PDK inhibitor	Glucose metabolism, mitochondrial function	Colorectal	Clinical trials^[113]^
Etomoxir	CPT1 inhibitor	Fatty acid oxidation	Colorectal	Preclinical^[114]^
CB-839	Glutaminase inhibitor	Glutamine metabolism	Colorectal, pancreatic	Clinical trials^[115]^
IACS-010759	Complex I inhibitor	OXPHOS	Pancreatic	Clinical trials^[116]^
Enasidenib	IDH2 inhibitor	TCA cycle	Cholangiocarcinoma	FDA approved^[117]^
Ivosidenib	IDH1 inhibitor	TCA cycle	Cholangiocarcinoma	FDA approved^[118]^
Statins (e.g., Atorvastatin)	HMG-CoA reductase inhibitor	Cholesterol metabolism	Colorectal	Clinical trials^[119]^
Orlistat	FASN inhibitor	Lipid metabolism	Colorectal	Preclinical^[120]^

FDA: Food and Drug Administration; 5-FU: 5-fluorouracil; VEGF: vascular endothelial growth factor; TCA cycle: tricarboxylic acid cycle: PDK: pyruvate dehydrogenase kinases; IACS: international association of classification societies; IDH1: isocitrate dehydrogenase 1; IDH2: isocitrate dehydrogenase-2; HMG-CoA: hydroxymethylglutaryl-Coenzyme; OXPHOS: oxidative phosphorylation; FASN: fatty acid synthase.

## THERAPEUTIC IMPLICATIONS

The therapeutic implications of targeting immune cell metabolism in GI cancers represent a promising frontier in overcoming drug resistance and enhancing treatment efficacy^[121]^. It becomes increasingly clear that modulating metabolic pathways in TAMs, MDSCs, and Tregs could significantly impact the TME and augment antitumor immune responses^[122]^. This approach offers a novel strategy to complement and potentially synergize with existing therapies, including chemotherapy, targeted therapies, and immunotherapies^[122]^. By altering the metabolic landscape within the tumor, we may be able to reprogram immunosuppressive cells toward more antitumor phenotypes, reinvigorate exhausted T cells, and create a more favorable environment for effective immune surveillance^[123]^. Furthermore, targeting specific metabolic vulnerabilities of these immune cell populations could selectively modulate their function without broadly suppressing the immune system, potentially reducing off-target effects and improving the overall therapeutic index^[124]^. Additionally, it is crucial to consider the heterogeneity of GI cancers and the dynamic nature of the TME, necessitating a personalized approach to metabolic modulation that takes into account the unique metabolic profile of each patient’s tumor and immune landscape. The potential therapeutic agents targeting immune cell metabolism in GI Cancers are shown in Table 4.

**Table 4:** Potential therapeutic agents targeting immune cell metabolism in GI cancers

**Therapeutic agent**	**Targeted metabolic pathway**	**Preclinical/Clinical status**	**Combination potential**	**References**
Etomoxir	FAO	Preclinical	With immunotherapy (e.g., PD-1 inhibitors)	[125]
Metformin	Glycolysis, AMPK activation	Clinical trials ongoing	With chemotherapy, immunotherapy	[126,127]
CB-839	Glutaminase inhibition	Phase I/II trials	With chemotherapy, targeted therapies	[128]
IACS-010759	OXPHOS inhibition	Phase I trials	With immunotherapy	[129,130]
Enasidenib	IDH2 inhibition	FDA-approved for AML, preclinical for GI cancers	With chemotherapy	[131]
Dichloroacetate	Pyruvate dehydrogenase kinase inhibition	Preclinical	With glycolysis inhibitors	[132]
DON	Glutamine metabolism	Preclinical	With immunotherapy	[133]
BPTES	Glutaminase inhibition	Preclinical	With chemotherapy	[134]
2-deoxyglucose	Glycolysis	Preclinical	With OXPHOS inhibitors	[135]
Arginine depletion (e.g., ADI-PEG20)	Arginine metabolism	Phase II/III trials	With chemotherapy, immunotherapy	[136]

ADI-PEG20: Pegylated arginine deiminase; OXPHOS: oxidative phosphorylation; BPTES: selective allosteric glutaminase (GLS1) inhibitor, DON: deoxynivalenol, CB-839: telaglenastat, active glutaminase 1 (GLS1) inhibitor, FAO: fatty acid oxidation; GI: gastrointestinal cancers; FDA: Food and Drug Administration; DON: 6-Diazo-5-oxo-L-norleucine.

## TARGETING FATTY ACID METABOLISM

**Figure 1:**
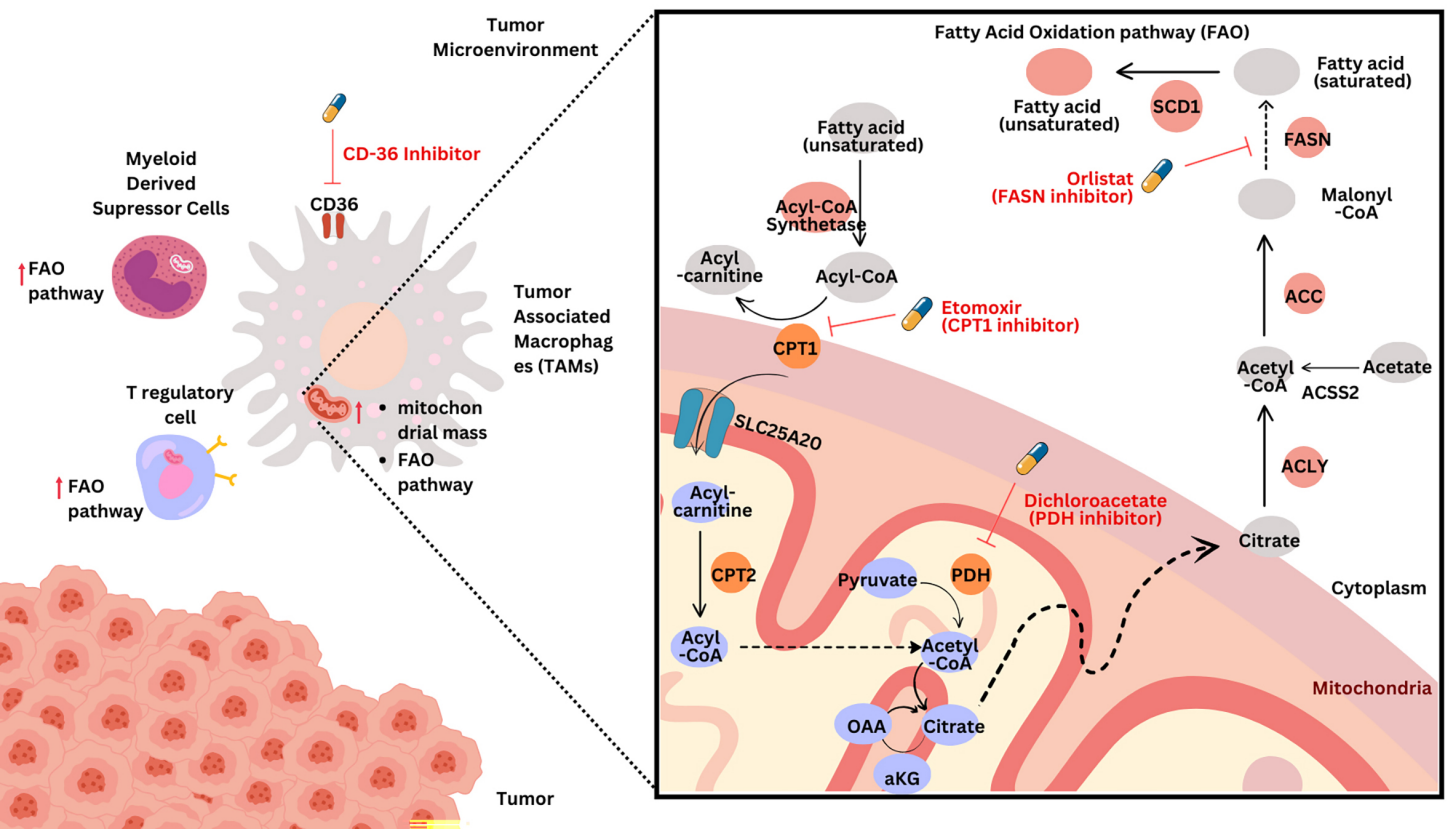
Fatty acid oxidation metabolic pathways in immune cells contributing to drug resistance in GI cancers. GI: Gastrointestinal cancers; ACC: Acetyl-CoA carboxylase; FASN: fatty acid synthase.

Fatty acid metabolism plays a crucial role in shaping the function and survival of immunosuppressive cells within the TME of GICs^[30]^. Targeting this metabolic pathway offers promising strategies to overcome drug resistance and enhance the efficacy of existing therapies. Inhibitors of FAO have emerged as potential agents to modulate the immunosuppressive functions of TAMs and Tregs^[137]^. These cell populations rely heavily on FAO to support their survival and differentiation, distinguishing them from effector T cells that primarily depend on glycolysis^[138]^. By inhibiting FAO, it may be possible to selectively impair the function of TAMs and Tregs without broadly suppressing the immune system. Studies have shown that Tregs preferentially activate OXPHOS and reduce tumor glucose uptake, making them more resilient in the glucose-depleted TME^[139]^. The FAO metabolic pathway contributing to drug resistance in GI cancers is described in Figure 1.

Disrupting this metabolic adaptation through FAO inhibition could potentially reduce the suppressive capacity of Tregs and enhance antitumor immunity. Several FAO inhibitors are currently being investigated for their potential to synergize with existing cancer therapies^[140]^. Etomoxir, a carnitine palmitoyltransferase I (CPT1) inhibitor, has shown promise in preclinical studies by reducing the immunosuppressive function of Tregs and enhancing the efficacy of immune checkpoint inhibitors^[133]^. Similarly, targeting the CD36 fatty acid transporter with monoclonal antibodies has been demonstrated to effectively block fatty acid uptake and lipid metabolism in tumor-infiltrating Tregs, hindering their accumulation and function in melanoma models without causing systemic loss of Tregs^[141]^.

FAS inhibitors represent another promising avenue for targeting immunosuppressive cells, particularly MDSCs^[142]^. MDSCs are known to accumulate in the TME and contribute significantly to immune evasion and drug resistance in GI cancers^[143]^. These cells produce immunosuppressive cytokines such as IL-10 and transforming growth factor-beta, which inhibit the activation and proliferation of T cells and NK cells, thereby creating an environment conducive to tumor growth^[144]^. Additionally, these cells deplete critical nutrients like arginine and cysteine from the TME through the expression of enzymes such as arginase-1 and IDO, further impairing T cell function^[145]^. They also directly inhibit immune responses by producing reactive oxygen species (ROS) and nitric oxide (NO), which can induce apoptosis in activated T cells. They also contribute to drug resistance by protecting tumor cells from the cytotoxic effects of chemotherapy and diminishing the effectiveness of immunotherapies, such as immune checkpoint inhibitors, by suppressing T cell activation. Furthermore, MDSCs interact with cancer stem cells, contributing to their maintenance and expansion, which is a key factor in both recurrence and resistance to therapy in GI cancers. This environment not only facilitates initial tumor growth but also promotes tumor recurrence following treatment by allowing residual cancer cells to survive unchecked^[146]^.

By targeting FAS, it may be possible to impair MDSC survival and function, thereby restoring the immune system’s ability to combat cancer cells^[147]^. For example, 5-tetradecyloxy-2-furoic acid (TOFA) - an inhibitor of Acetyl-CoA carboxylase (ACC) - has been shown to disrupt FAS in tumor-infiltrating immune cells, leading to regression of murine hepatocellular carcinoma^[148]^. However, in some studies the observed therapeutic effects may result from direct drug toxicity rather than specific interference with immunometabolism, highlighting the need for careful evaluation of these approaches. The potential of targeting fatty acid metabolism extends beyond its direct effects on immunosuppressive cells^[149]^. Modulating this pathway may also influence the overall metabolic landscape of the TME, potentially creating a more favorable environment for antitumor immune responses^[149]^. Inhibiting FAS in cancer cells could reduce the availability of lipid mediators that promote the recruitment and differentiation of immunosuppressive cell populations^[150]^. While inhibiting FAO may impair Treg function, it could also affect the formation of long-term memory T cells, which rely on FAO for their development and persistence^[151]^. Therefore, strategies that combine metabolic modulation with other immunotherapeutic approaches, such as checkpoint inhibitors or adoptive cell therapies, may offer the most promising path forward. Targeting fatty acid metabolism in immunosuppressive cells within the TME has emerged as a promising strategy to enhance antitumor immunity and overcome drug resistance in GICs^[152]^. This approach focuses on two key aspects: inhibiting FAO in TAMs and Tregs, and targeting FAS in MDSCs^[83,153]^. Inhibitors of FAO have shown potential in reducing the immunosuppressive functions of TAMs and Tregs, thereby enhancing the antitumor activity of existing therapies^[154]^. In human and mouse hepatocellular carcinoma tissues, the inhibition of receptor-interacting protein kinase 3 (RIPK3) in TAM using inhibitors like Decitabine, a DNA methyltransferase inhibitor, inhibits caspase-1-mediated cleavage of PPAR that promotes FAO and causes a reversal of the pro-tumor phenotype of TAMs that is M2 phenotypes^[155]^.

In one study, etomoxir treatment of tumor-bearing mice resulted in a T-cell-dependent inhibition of tumor growth and enhanced the antitumor effect of low-dose chemotherapy and adoptive cellular therapy^[156]^. Targeting the CD36 fatty acid transporter with monoclonal antibodies has been shown to effectively block fatty acid uptake and lipid metabolism in tumor-infiltrating Tregs^[157]^. CD36 is a transmembrane glycoprotein that plays a significant role in the TME by mediating lipid uptake and influencing immune responses^[158]^. In GI, several specific cell types express CD36, contributing to tumor progression and immune evasion. Notably, TAMs exhibit high levels of CD36 expression, which facilitates their uptake of lipid-rich extracellular vesicles released by tumor cells^[159]^. This lipid uptake enhances the metabolic reprogramming of TAMs, promoting their tumor-promoting activities and contributing to an immunosuppressive environment^[160]^. Additionally, cancer-associated fibroblasts can express CD36, although its expression tends to decrease as these cells transform from normal fibroblasts^[161]^. In some contexts, CAFs with low CD36 expression may produce more extracellular matrix components, which can support tumor growth and metastasis. Furthermore, tumor cells themselves can also express CD36, which is associated with increased metastatic potential and the induction of EMT, a process that enhances cell migration and invasion^[162]^. This approach hindered the accumulation and function of Tregs in melanoma models without causing systemic loss of Tregs. Notably, studies have demonstrated the efficacy of TOFA in impairing FAS in tumor-infiltrating immune cells and thus inducing regression of murine hepatocellular carcinoma^[163]^. As mentioned, however, these therapeutic benefits may arise from direct drug toxicity rather than targeted immunometabolic effects. Targeting FAS in MDSCs represents another promising avenue for modulating the immunosuppressive TME^[164]^. Fatty acid synthase (FASN) inhibitors have been shown to impair MDSC function and survival in various cancer models^[120]^. The FASN inhibitor C75 has been demonstrated to reduce MDSC accumulation and enhance antitumor immunity in breast cancer models^[165]^. ACC inhibitors, which target a key enzyme in the FAS pathway, have shown potential in modulating MDSC function^[166]^. While not specifically studied in MDSCs, ACC inhibitors have demonstrated antitumor effects in various cancer types and may have implications for MDSC metabolism. The potential of targeting fatty acid metabolism extends beyond its direct effects on immunosuppressive cells^[167]^. Modulating these pathways may also influence the overall metabolic landscape of the TME, potentially creating a more favorable environment for antitumor immune responses. Inhibiting FAS in cancer cells could reduce the availability of lipid mediators that promote the recruitment and differentiation of immunosuppressive cell populations^[168]^.

## MODULATING GLYCOLYSIS

**Figure 2:**
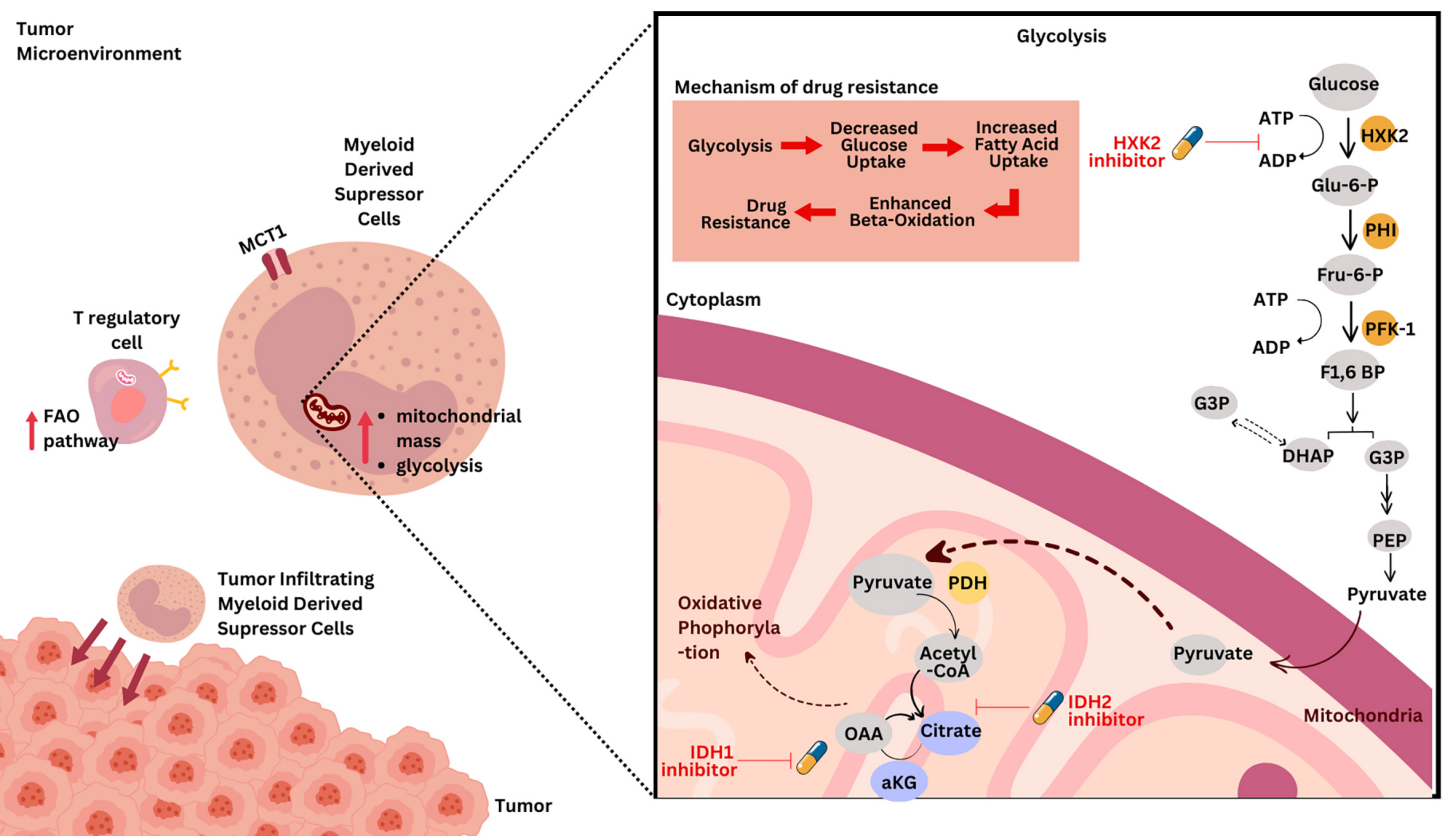
Glycolysis pathways in immune cells and their role in mediating drug resistance in GI cancers. This figure illustrates the critical metabolic pathways in MDSCs, TAMs, and Tregs that contribute to drug resistance in gastrointestinal cancers. These immune cells undergo metabolic reprogramming, promoting an immunosuppressive TME that supports cancer progression and reduces the efficacy of therapies. MDSCs: Myeloid-derived suppressor cells; TAMs: tumor-associated macrophages; Tregs: regulatory T cells; TME: tumor microenvironment.

Modulating glycolysis in MDSCs and other immunosuppressive cells has emerged as a promising strategy to reduce their ability to support tumor growth and drug resistance in GICs^[169]^. Figure 2 depicts the glycolysis metabolic pathway (lower panel) in immune cells, highlighting its role in contributing to drug resistance in GI cancers. Glycolysis inhibitors can decrease the energy supply of these cells, potentially impairing their immunosuppressive functions and enhancing antitumor immunity^[170]^. Several studies have demonstrated the importance of glycolysis in MDSC function and survival. MDSCs have been shown to rely heavily on glycolysis for their energy needs, particularly in the TME. *In vitro*-generated MDSCs display increased glycolysis, glutaminolysis, and TCA cycle activity^[171]^. This metabolic profile supports their rapid proliferation and immunosuppressive functions. Inhibition of glycolysis using compounds like 2-deoxy-D-glucose (2DG) has been shown to suppress the differentiation of MDSCs from precursor cells^[172]^. In one study, 2DG treatment decreased the survival of MDSCs stimulated with C. tropicalis and markedly impaired the expression of immunosuppressive factors like iNOS, COX2, and NOX2^[173]^.

Interestingly, MDSCs display metabolic plasticity depending on their tissue origin and microenvironment. While peripheral MDSCs rely more on glycolysis, tumor-infiltrating MDSCs have been observed to have increased mitochondrial mass and preferentially use FAO over glycolysis as a primary energy source^[156]^. This highlights the importance of considering the specific metabolic profile of MDSCs in different contexts when designing therapeutic strategies. Recent research has identified methylglyoxal, a glycolytic by-product, as a more specific marker for MDSCs^[169]^. This compound may play a key role in the suppression of T effector function. Neutralization of methylglyoxal’s dicarbonyl activity has shown promise in improving the efficacy of cancer immunotherapy^[83]^. Targeting glycolysis in MDSCs may be particularly effective when combined with other therapeutic approaches. Inhibition of glycolysis could potentially enhance the efficacy of immune checkpoint inhibitors by reducing the immunosuppressive capacity of MDSCs in the TME^[174]^. However, it is important to note that modulating glycolysis can have complex effects on the TME. While reducing glycolysis in MDSCs may be beneficial, it is crucial to consider that effector T cells also rely heavily on glycolysis for their proliferation and function^[65]^. Therefore, systemic inhibition of glycolysis could potentially impair antitumor immune responses. The use of glycolysis inhibitors may alter the metabolic competition between tumor cells, MDSCs, and effector immune cells in the TME. This could potentially create more favorable conditions for antitumor immunity if carefully modulated^[175]^. As MDSCs demonstrate metabolic plasticity, inhibiting glycolysis alone may lead to compensatory upregulation of alternative metabolic pathways, such as FAO. Therefore, combination strategies targeting multiple metabolic pathways may be necessary for optimal therapeutic efficacy^[176]^.

## COMBINATION THERAPIES

Combination therapies involving metabolic inhibitors and standard cancer treatments have emerged as a promising strategy to overcome drug resistance and improve patient outcomes in GICs^[177]^. This approach aims to exploit the metabolic vulnerabilities of cancer cells while simultaneously enhancing the efficacy of existing therapies. Several preclinical studies and early clinical trials have demonstrated the potential of such combinations. They have shown synergistic effects when combining glycolysis inhibitors with traditional chemotherapeutic agents^[178]^. The combination of 2DG with cisplatin or doxorubicin has demonstrated enhanced antitumor activity in various cancer models, including gastric cancer^[179]^. The rationale behind this combination is that metabolic inhibition can sensitize cancer cells to DNA-damaging agents by depleting their energy resources and impairing DNA repair mechanisms. In a mouse model of colorectal cancer, the FAO inhibitor etomoxir showed synergistic effects when combined with low-dose cyclophosphamide^[180]^. This combination resulted in enhanced T-cell-dependent inhibition of tumor growth, suggesting that modulating immune cell metabolism can potentiate the effects of chemotherapy. Further, Decitabine and ionizing radiation in combination improved the immunogenicity and susceptibility of tumor cells to immune cells by upregulating the expression of major histocompatibility complex (MHC) class I, natural-killer group 2, member D (NKG2D) ligands, and co-stimulatory molecules^[181]^.

Combining metabolic inhibitors with targeted therapies has shown promise in overcoming resistance mechanisms^[182]^. In HER2-positive breast cancer models, inhibition of glutaminase (GLS) using the compound CB-839 (telaglenastat) enhanced the efficacy of trastuzumab. While this study focused on breast cancer, it provides a rationale for exploring similar combinations in HER2-positive gastric cancers^[183]^. The glycolysis pathway can be targeted to reduce the drug resistance. For instance, it has been reported that the resistance to gemcitabine, a synthetic pyrimidine-nucleoside prodrug (chemotherapy drug), reduced on treatment along with LDH-A inhibitors (N-hydroxyindole-2-carboxylates; NHI-1,NH-2) and GLUT-1 inhibitors (PGL13, PGL14, salicylketoxime derivatives) in malignant mesothelioma (MM) that reverse the cell’s metabolism from glycolysis to OXOPHOS pathways^[184]^. The resistance to OXPHOS inhibitors can be improved with a combination of therapies such as Mitochondria-targeted atovaquone (Mito-ATO) in MDSCs and Tregs with PD-1 blockade and other immune checkpoint inhibitors^[185]^.

One of the most exciting areas of combination therapy involves pairing metabolic inhibitors with immune checkpoint inhibitors. Preclinical trials have demonstrated that the combination of MCT1 inhibitor AZD3965 and anti-PD-1 therapy can reduce lactate secretion into the TME, decrease the infiltration of exhausted PD-1+ Tim-3+ T cells in solid tumors, and improve antitumor immunity^[186]^. In a mouse model of hepatocellular carcinoma, inhibition of MCT4 led to reduced CD8+ T cell exhaustion and enhanced antitumor immune responses to immune checkpoint inhibitors^[187]^. This suggests that modulating the metabolic landscape of the TME can create more favorable conditions for immunotherapy to be effective. Given the metabolic plasticity of cancer cells, simultaneously targeting multiple metabolic pathways may be necessary for optimal therapeutic efficacy^[188]^. Combining inhibitors of glycolysis (such as 2DG) with inhibitors of glutaminolysis (CB-839) has shown synergistic effects in preclinical models of various cancers^[129]^. This approach aims to prevent compensatory upregulation of alternative metabolic pathways when a single pathway is inhibited. While many of these combination strategies have shown promise in preclinical studies, their efficacy in human patients remains to be fully established. Several clinical trials are currently underway to evaluate the safety and efficacy of metabolic inhibitors in combination with standard therapies. A phase I/II trial (NCT01791595) is evaluating the MCT1 inhibitor AZD3965 in combination with various cancer therapies, including immune checkpoint inhibitors^[189]^. The GLS inhibitor telaglenastat (CB-839) is being tested in combination with standard chemotherapies and targeted therapies in various solid tumors, including colorectal cancer (NCT02861300)^[190]^. Trials combining IDO1 inhibitors with immune checkpoint inhibitors are ongoing, despite initial setbacks in melanoma studies. These trials aim to determine if IDO1 inhibition can enhance the efficacy of immunotherapy in GICs^[191]^.

## FUTURE DIRECTIONS

The importance of exploring future directions in the field of metabolic modulation of immune cells in GI cancers is underscored by several critical factors. Persistent drug resistance remains a significant challenge, limiting the efficacy of existing therapies and hindering patient outcomes^[192]^. Metabolic modulation represents a promising approach to overcome this resistance by targeting the underlying metabolic mechanisms that support tumor survival and immune evasion. Additionally, the complexity of the TME presents a dynamic ecosystem where interactions between cancer cells, immune cells, and stromal components occur through various metabolic pathways^[193]^. Our current understanding of these intricate interactions is still limited, necessitating further research to fully exploit the therapeutic potential of metabolic interventions. Furthermore, the heterogeneity of immune responses highlights the variability in metabolic states among patients and within different tumor regions, emphasizing the need for personalized approaches and sophisticated methods to characterize specific metabolic profiles. The integration of metabolic modulation with existing therapies, such as immunotherapies and targeted treatments, also warrants exploration to optimize combination strategies and identify synergistic effects. Moreover, the identification of reliable metabolites is essential for patient stratification, treatment selection, and monitoring therapeutic responses, which could significantly enhance treatment precision, and thus reduce off-target effects. Technological advancements in metabolomics, single-cell analysis, and *in vivo* imaging are opening new avenues for studying cancer metabolism at unprecedented resolution; harnessing these technologies will be vital for deepening our understanding of the metabolic landscape in GI cancers^[194,195]^. Finally, addressing the long-term effects of metabolic interventions on tumor progression and overall patient health is critical, as extended follow-up studies are necessary to assess response durability and identify any potential long-term implications. Research in the metabolic modulation of immune cells is rapidly evolving, with a promising impact on overcoming drug resistance in GI cancers. As our understanding of the complex interplay between tumor metabolism, immune cell function, and the TME deepens, several key areas emerge as critical focuses for future research and clinical development.

## BIOMARKER DEVELOPMENT

The identification and validation of reliable biomarkers that can predict response to metabolic therapies stand as a cornerstone for progress in cancer immunometabolism^[196]^. Future studies must prioritize the development of comprehensive metabolic profiling techniques to identify unique signatures associated with response to specific metabolic inhibitors. This endeavor will likely involve a multifaceted approach, combining advanced metabolomics techniques with cutting-edge imaging modalities and molecular profiling^[197]^. Researchers should focus on characterizing the metabolic states of both tumor cells and various immune cell populations within the TME, potentially revealing novel predictive markers for immunotherapy response^[198]^. Single-cell metabolomics and flow cytometry techniques need to be employed to assess the metabolic activity of specific immune cell subsets, providing invaluable insights into the heterogeneity of cellular metabolism within the tumor ecosystem^[199]^. The exploration of circulating biomarkers through liquid biopsies presents a particularly attractive avenue, offering the potential for non-invasive, real-time monitoring of treatment efficacy. This approach could revolutionize patient care by allowing for dynamic adjustment of treatment strategies based on evolving metabolic profiles.

Additionally, the advancement of metabolic imaging techniques, such as hyperpolarized MRI and PET scans with innovative tracers, may provide valuable insights into the dynamic metabolic landscape of tumors and immune cells *in vivo*^[200]^. These imaging biomarkers could offer a non-invasive means of visualizing and quantifying metabolic activity, potentially guiding treatment decisions and monitoring response to therapy^[201]^. The use of microRNA as a biomarker for GIC drug resistance is also on the rise^[202]^. As these biomarker discovery efforts progress, it will be crucial to conduct large-scale, multi-center validation studies to ensure the robustness and generalizability of findings across diverse patient populations and cancer types. The integration of artificial intelligence and machine learning algorithms to analyze complex metabolomic, genomic, and clinical data may further enhance our ability to identify and interpret relevant biomarkers, ultimately leading to more accurate prediction of treatment outcomes and personalized therapeutic strategies^[203]^.

## CLINICAL TRIALS

The current state of research and development in the field of metabolic reprogramming highlights its potential as a transformative approach in cancer therapy. Ongoing clinical trials, promising preclinical findings, and innovative technologies are paving the way for new therapeutic strategies aimed at overcoming drug resistance and enhancing treatment efficacy across various cancer types. Future clinical research should prioritize investigating synergistic effects between metabolic modulators and established treatment modalities, including immune checkpoint inhibitors, chemotherapy, and targeted therapies. For instance, targeting metabolic pathways that promote T cell exhaustion could enhance the effectiveness of CAR-T cell therapies, as suggested by findings that manipulating T cell metabolism can reinvigorate their antitumor activity^[13]^. Recent preclinical studies have shown that activating the pentose phosphate pathway (PPP) in T cells can improve their effectiveness against tumors when combined with immune checkpoint inhibitors^[204]^. Researchers at Weill Cornell Medicine are exploring this approach further, with plans to develop agents that induce T cell reprogramming for future clinical trials, aiming to enhance patient responses to therapies such as PD-1 blockers by maintaining T cells in a more effective precursor state. Clinical trials are underway to evaluate the efficacy of GLS inhibitors, such as CB-839, which target glutamine metabolism in various cancers, including breast and renal cancers^[205]^. These inhibitors aim to disrupt the metabolic flexibility that cancer cells rely on for survival and resistance to conventional therapies. These studies must carefully consider optimal dosing schedules and sequences to maximize therapeutic benefits while minimizing potential toxicities^[206]^. The incorporation of biomarker-based patient selection criteria will be essential in enriching trial populations with those most likely to benefit from metabolic interventions, potentially leading to more efficient and informative clinical outcomes. This approach could significantly reduce the time and resources required to bring effective therapies to market, while also sparing patients from unnecessary exposure to potentially ineffective treatments. Moreover, the development and validation of novel clinical trial endpoints that reflect the unique mechanisms of action of metabolic therapies will be crucial^[207]^. These may include measures of immune cell function, metabolic activity, or changes in TME composition, providing a more comprehensive assessment of treatment efficacy beyond traditional measures of tumor size or progression-free survival^[208]^.

Furthermore, research has identified phosphoenolpyruvate carboxykinase 1 (PCK1) as a critical regulator of tumor metabolism and proliferation. Studies have demonstrated that targeting PCK1 can significantly impair cancer cell growth and metastasis^[209]^. The therapeutic potential of PCK1 inhibitors is currently being explored in various malignancies, including colorectal and renal cancers.

Long-term follow-up studies will also be necessary to assess the durability of responses to metabolic therapies and identify any potential long-term effects on immune function or overall health. As these clinical trials progress, researchers must remain vigilant in monitoring for potential combination toxicities and developing strategies to mitigate such risks. This may involve the development of innovative trial designs, such as adaptive protocols that allow for real-time adjustment of treatment regimens based on observed efficacy and toxicity profiles. Additionally, the inclusion of quality-of-life measures and patient-reported outcomes in clinical trials will be essential to fully understand the impact of metabolic therapies on patient well-being and to guide decision making in clinical practice^[210]^.

## PERSONALIZED MEDICINE

The ultimate goal of research in cancer immunometabolism is the development of personalized therapeutic approaches tailored to the unique metabolic profile of each patient’s tumor and immune landscape^[211]^. Achieving this ambitious objective will require significant advancements in several interconnected areas. First, the establishment of standardized methods for comprehensive metabolic profiling of tumor biopsies, including assessment of nutrient availability, metabolite concentrations, and enzyme activities within the TME, will be essential^[212]^. This profiling should be complemented by the integration of artificial intelligence and machine learning algorithms capable of analyzing complex metabolomic, genomic, and clinical data to predict optimal treatment strategies for individual patients. The development of sophisticated *ex vivo* models, such as patient-derived organoids or “tumor-on-a-chip” systems that accurately recapitulate the metabolic landscape of individual tumors, could enable rapid testing of various metabolic interventions before administration to patients^[213]^. These models may serve as powerful tools for predicting treatment response and identifying potential resistance mechanisms, allowing for more informed and personalized treatment decisions. Furthermore, the exploration of adaptive treatment strategies that adjust metabolic interventions based on real-time monitoring of tumor and immune cell metabolism represents an exciting frontier in personalized cancer therapy.

Advanced metabolomic techniques could be used to create comprehensive metabolic profiles of individual tumors. Mass spectrometry-based metabolomics might reveal specific alterations in glucose, glutamine, or fatty acid metabolism unique to a patient’s GI cancer^[214]^. This information could then guide the selection of metabolic inhibitors or activators most likely to be effective for that particular tumor^[215]^. Single-cell metabolomics technologies may allow for detailed characterization of immune cell metabolic states within the TME^[216]^. This could help identify patients whose tumors have particularly immunosuppressive metabolic profiles, making them candidates for combination therapies that couple immune checkpoint inhibitors with metabolic modulators to reinvigorate exhausted T cells. The development of blood-based tests to detect metabolic biomarkers could enable real-time monitoring of treatment response and early detection of resistance. Circulating metabolites or exosomes containing metabolic enzymes might serve as indicators of tumor metabolism, allowing for dynamic adjustment of treatment strategies^[217]^.

Genetic variations affecting drug metabolism or target proteins could influence the efficacy and toxicity of metabolic therapies^[218]^. Screening for these variations could help optimize dosing and predict potential side effects individually^[219]^. Polymorphisms in genes encoding metabolic enzymes like IDH1/2 might affect response to IDH inhibitors in certain GI cancers^[220]^. The development of patient-derived organoid models that recapitulate the metabolic landscape of individual tumors could serve as powerful tools for personalized drug screening^[221]^. These “mini-tumors” grown in the lab could be used to test various metabolic interventions before administering them to the patient, potentially improving treatment outcomes and reducing unnecessary toxicity. Additionally, adaptive treatment protocols that adjust metabolic interventions based on real-time monitoring of tumor metabolism are emerging as a valuable approach^[222]^. This could involve periodic reassessment of tumor metabolic profiles and therapy adjustment to counteract emerging resistance mechanisms^[223]^. Combining metabolomic data with genomic, transcriptomic, and proteomic information could provide a more comprehensive view of tumor biology. Machine learning algorithms could then analyze this complex data and predict the most effective personalized treatment strategies^[224]^. Given the growing recognition of the gut microbiome’s influence on metabolism and immune function, personalized approaches might incorporate microbiome analysis to guide dietary interventions or the use of specific probiotics to optimize the metabolic environment for antitumor immunity^[225]^.

As we advance toward truly personalized metabolic therapies, it will be crucial to consider the broader context of each patient’s overall health, lifestyle, and environmental factors that may influence tumor metabolism and immune function. This holistic approach may involve integrating data from wearable devices, dietary assessments, and microbiome analyses to create a comprehensive metabolic profile for each patient. Additionally, the development of targeted drug delivery systems that can selectively modulate metabolism in specific cell populations within the TME may further enhance the precision and efficacy of personalized treatments.

## DISCUSSION

The intricate interplay between metabolic reprogramming and immune cell function within the TME has emerged as a critical factor in the development of drug resistance in GI cancers^[226]^. Increasing evidence suggests that metabolic adaptations in neutrophils, T helper cells, and innate lymphoid cells (ILCs) significantly influence immunotherapy outcomes and other treatment modalities^[227]^. This emerging understanding highlights the importance of not only targeting cancer cell metabolism but also considering the metabolic alterations occurring within immune cells. Neutrophils, T helper cells, and ILCs undergo significant metabolic shifts in response to the TME, which can influence their functionality and effectiveness in antitumor responses^[228]^. The metabolic adaptations observed in both cancer cells and immune cells within the TME create a unique landscape that often favors tumor growth and immune evasion. Cancer cells, through their altered metabolism, not only sustain their own proliferation but also reshape the TME, creating nutrient-depleted, hypoxic, and acidic conditions that impair the function of antitumor immune cells^[229]^. Simultaneously, immune cells undergo their own metabolic reprogramming, often adopting phenotypes that are less effective in combating the tumor or even actively suppress antitumor immunity. Our exploration of this metabolic crossroads has revealed several key insights. First, the competition for nutrients between cancer cells and immune cells significantly influences the efficacy of antitumor responses. Tumor cells often outcompete T cells and other effector immune cells for glucose and other essential metabolites, leading to impaired immune function^[230]^. Second, the accumulation of metabolic by-products in the TME, such as lactate and kynurenine, can directly suppress immune cell activity and promote immunosuppressive cell types like Tregs and M2 macrophages^[231]^. Furthermore, we have uncovered how specific metabolic pathways, such as FAO, glycolysis, OXOPHOS, and amino acid metabolism, play crucial roles in determining immune cell fate and function within the TME.

As we look to the future, the development of strategies to modulate these metabolic pathways presents a promising avenue for enhancing the efficacy of existing cancer therapies, particularly immunotherapies. Targeting cancer metabolism as a therapeutic strategy presents significant challenges due to the rapid adaptability of cancer cells. One major limitation is the phenomenon of metabolic reprogramming, where cancer cells can swiftly alter their metabolic pathways in response to therapeutic interventions^[232]^. This adaptability often allows them to evade targeted therapies that aim to exploit specific metabolic dependencies. For instance, while some therapies may initially show effectiveness by targeting unique metabolic traits of cancer cells, these cells can quickly shift their metabolism to utilize alternative pathways, rendering the treatment ineffective over time. Moreover, the intricate interplay between cancer cells and their surrounding TME complicates therapeutic targeting. The metabolic activities of stromal cells, immune cells, and cancer-associated fibroblasts contribute to a dynamic metabolic network that supports tumor growth and survival. As a result, therapies that target cancer cell metabolism must also consider the metabolic contributions of these non-cancerous cells, which can further complicate treatment outcomes and lead to resistance^[233]^. Additionally, the heterogeneity observed among different tumor types poses another challenge. Variability in metabolic profiles not only exists between distinct cancers but also within different regions of the same tumor. This heterogeneity can lead to differential responses to metabolic therapies, making it difficult to develop universally effective treatments. Consequently, while targeting metabolism holds promise as a cancer therapy strategy, these limitations highlight the need for more refined approaches that account for the rapid adaptability and complex interactions within the tumor ecosystem^[234]^.

By reprogramming the metabolism of immune cells or altering the metabolic landscape of the TME, we may be able to reinvigorate exhausted T cells, redirect immunosuppressive cell types toward antitumor phenotypes, and ultimately overcome drug resistance^[235]^. However, translating these insights into effective therapies will require a multifaceted approach. Continued basic research is essential to further elucidate the complex metabolic networks at play within the TME^[236]^. Concurrently, the development of novel metabolic inhibitors and activators, as well as innovative drug delivery systems to target specific cell populations, will be crucial. Clinical trials investigating combination therapies that pair metabolic modulators with existing treatments, such as immune checkpoint inhibitors or targeted therapies, will be vital in determining the most effective strategies for different GI cancer types and patient subgroups^[237]^. Additionally, the identification and validation of metabolic biomarkers will be essential for patient stratification and monitoring treatment response. The principles uncovered in GI cancers may have far-reaching applications across various cancer types, potentially revolutionizing our approach to cancer treatment. Metabolic reprogramming is not unique to GI cancers; it is a widespread phenomenon observed in many cancers, including breast, lung, and prostate cancers^[238]^. Studies have shown that breast cancer cells often exhibit increased glycolysis and altered lipid metabolism, similar to findings in GICs^[239]^. Since the TME plays a crucial role in influencing cancer cell metabolism and therapeutic responses, many solid tumors, including pancreatic and renal cancers, experience hypoxic conditions that drive metabolic reprogramming. Hypoxia-inducible factors (HIFs) regulate glucose metabolism under low oxygen conditions, promoting glycolysis and lactate production. Inhibitors targeting HIF pathways are being investigated as potential therapeutic strategies to counteract hypoxia-induced resistance^[240]^.

The interplay between epigenetic modifications and metabolic reprogramming is increasingly recognized as a critical factor in cancer progression^[241]^. Using the commonalities between GI cancers and other cancer types, DNA methylation alterations can be linked to changes in glucose and lipid metabolism in breast and prostate cancers^[242]^. The principles of metabolic reprogramming can inform the development of combination therapies that target both cancer cell metabolism and the TME. Glutaminolysis is a common metabolic pathway exploited by many tumors, including glioblastoma and breast cancer. GLS inhibitors like CB839 are being evaluated in clinical trials for their ability to disrupt glutamine metabolism and enhance the efficacy of standard chemotherapeutics^[243]^. Along with this, the mTOR signaling pathway, which regulates cellular metabolism and growth, is frequently hyperactivated in various cancers. mTOR inhibitors have shown promise in preclinical studies for treating breast cancer and renal cell carcinoma by targeting metabolic pathways that promote resistance^[244]^.

## DECLARATIONS

### Copyright

© The Author(s) 2025.
